# Review on Cross Talk between Neurotransmitters and Neuroinflammation in Striatum and Cerebellum in the Mediation of Motor Behaviour

**DOI:** 10.1155/2019/1767203

**Published:** 2019-11-14

**Authors:** Dayang Yasmin Abg Abd Wahab, Chuang Huei Gau, Rahimah Zakaria, Mohan Kumar Muthu Karuppan, Badriya S. A-rahbi, Zuraidah Abdullah, Aziza Alrafiah, Jafri Malin Abdullah, Sangu Muthuraju

**Affiliations:** ^1^Department of Neurosciences and Brain and Behaviour Cluster, School of Medical Sciences, Universiti Sains Malaysia, Jalan Hospital Universiti Sains Malaysia, Kubang Kerian, 16150 Kota Bharu, Kelantan, Malaysia; ^2^Department of Physiology, School of Medical Sciences, Universiti Sains Malaysia, Jalan Hospital Universiti Sains Malaysia, Kubang Kerian, 16150 Kota Bharu, Kelantan, Malaysia; ^3^Department of Immunology and Nano-Medicine, Herbert Wertheim College of Medicine, Florida International University, 11200 SW 8th St, Miami, FL 33199, USA; ^4^Oman College of Health Sciences, Muscat, Oman; ^5^Department of Biomedical Sciences, School of Health Sciences, Universiti Sains Malaysia, Jalan Hospital Universiti Sains Malaysia, Kubang Kerian, 16150 Kota Bharu, Kelantan, Malaysia; ^6^Department of Medical Laboratory Technology, Faculty of Applied Medical Sciences, King AbdulAziz University, Jeddah, Saudi Arabia; ^7^Department of Pharmacological and Pharmaceutical Sciences, College of Pharmacy, University of Houston, Houston 77240, TX, USA

## Abstract

Neurological diseases particularly Alzheimer's disease (AD), Parkinson's disease (PD), stroke, and epilepsy are on the rise all around the world causing morbidity and mortality globally with a common symptom of gradual loss or impairment of motor behaviour. Striatum, which is a component of the basal ganglia, is involved in facilitating voluntary movement while the cerebellum is involved in the maintenance of balance and coordination of voluntary movements. Dopamine, serotonin, gamma-aminobutyric acid (GABA), and glutamate, to name a few, interact in regulating the excitation and inhibition of motor neurons. In another hand, interestingly, the motor loss associated with neurological diseases is possibly resulted from neuroinflammation induced by the neuroimmune system. Toll-like receptors (TLRs) are present in the central nervous system (CNS), specifically and primarily expressed in microglia and are also found on neurons and astrocytes, functioning mainly in the regulation of proinflammatory cytokine production. TLRs are always found to be associated or involved in the induction of neuroinflammation in neurodegenerative diseases. Activation of toll-like receptor 4 (TLR4) through TLR4 agonist, lipopolysaccharide (LPS), stimulation initiate a signaling cascade whereby the TLR4-LPS interaction has been found to result in physiological and behavioural changes including retardation of motor activity in the mouse model. TLR4 inhibitor TAK-242 was reflected in the reduction of the spinal cord pathology along with the motor improvement in ALS mouse. There is cross talk with neuroinflammation and neurochemicals. For example, TLR4 activation by LPS is noted to release proinflammatory cytokines, IL-1*β*, from microglia that subsequently suppresses GABA receptor activities at the postsynaptic site and reduces GABA synthesis at the presynaptic site. Glial glutamate transporter activities are also found to be suppressed, showing the association between TLR4 activation and the related neurotransmitters and corresponding receptors and transporters in the event of neuroinflammation. This review is helpful to understand the connection between neurotransmitter and neuroinflammation in striatum- and cerebellum-mediated motor behaviour.

## 1. Introduction

Neurological diseases particularly Alzheimer's disease (AD), Parkinson's disease (PD), stroke, and epilepsy are on the rise all around the world. As the second leading cause of morbidity and mortality globally, it has become one of the greatest threats to public health [1]. All the aforementioned diseases share a common symptom of gradual loss or impairment of motor behaviour. Based on the Global Burden of Disease Study 2015 [1], neurological diseases were listed and emerged as the top disease to cause 250.692 million disability-adjusted life years (DALYs), comprising 10.2% of global DALYs, and 9.399 million deaths, comprising 16.8% of global deaths, the second highest in terms of global deaths. Therefore, from the statistics, it is evident how critical it is to research on ways to alleviate the distress, the physical constraint that is affecting the people [1]. It has been noted that the motor loss associated with neurological diseases are possibly resulted from neuroinflammation induced by the neuroimmune system [2]. The immune system is one of the major functional components of the body that is responsible for the occurrence of neuroinflammation. One of the major, active components of the neuroimmune systems is the toll-like receptors (TLRs).

Toll-like receptors (TLRs) are always found to be associated or involved in the induction of neuroinflammation in neurodegenerative diseases. For example, deficiency of toll-like receptor 2 (TLR2) and toll-like receptor 4 (TLR4) in mice exhibits reduced levels of proinflammatory cytokines, resulting in milder clinical disease following traumatic brain injury. Subsequently, increased expression of TLR4 is also found in PD, AD, and amyotrophic lateral sclerosis (ALS) patients as well as in animal models [3].

TLR4 particularly has been demonstrated in various studies to have a significant causal relationship with motor dysfunction in neurodegenerative conditions. Activation of TLR4 through TLR4 agonist, lipopolysaccharide (LPS), stimulation initiate a signaling cascade whereby the TLR4-LPS interaction has been found to result in physiological and behavioural changes including retardation of motor activity in the mouse model [4]. LPS is a component of Gram-negative bacteria known to trigger inflammation, specifically known to activate TLR4. A number of studies reported the suppression of neuroinflammation through TLR4 inhibition which consequently minimized motor deficit in animal models involving neurodegenerative diseases as well as traumatic brain injury [5]. Numerous studies had demonstrated a significant relationship between TLR4 and motor impairments in neurodegenerative disorders. A recent study demonstrated an increase in motor impairments in a mice model as a result of TLR4 activation using monophosphoryl lipid A [6]. However, the involvement of the striatum and cerebellum has not been thoroughly reviewed yet.

Striatum, which is a component of the basal ganglia, is involved in facilitating voluntary movement while the cerebellum is involved in the maintenance of balance and coordination of voluntary movements. Both these structures work together with the cerebral cortex in mediating movements, and various neurotransmitters are involved in the circuitries involved in the process. Dopamine, serotonin, gamma-aminobutyric acid (GABA), and glutamate, to name a few, interact in regulating the excitation and inhibition of motor neurons. Studies had long demonstrated the involvement of such neurotransmitters in the proper functioning of motor neurons in the striatum and cerebellum [7]. In the motor system, serotonin (5-hydroxytryptamine, 5-HT) is found to either enhance or depress glutamate-mediated transmission as well as GABA-mediated transmission in structures controlling movement [8]. Additionally, TLR4 activation by LPS is noted to release proinflammatory cytokines, IL-1*β*, from microglia that subsequently suppresses GABA receptor activities at the postsynaptic site and reduces GABA synthesis at the presynaptic site. Glial glutamate transporter activities are also found to be suppressed, showing the association between TLR4 activation and the related neurotransmitters and corresponding receptors and transporters in the event of neuroinflammation [9]. The aim of this review is focusing on the role of microglia in the central nervous system, neuroinflammation in different kinds of neurological disease, how TLR involves in motor behaviour and mediating neuroinflammation signaling and dealing with what are important of striatum and cerebellum neurotransmitters in motor behaviour.

## 2. Neuroinflammation

Motor deficit emerged as a prominent feature or symptom in neurodegenerative diseases such as Parkinson's disease (PD), Alzheimer's disease (AD), and stroke. Motor deficits linked to the aforementioned diseases usually shows in the form of motor slowing (bradykinesia), gait and posture disturbances, rigidity, and resting tremor [10]. It has been noted that the motor loss associated with neurodegenerative diseases are possibly resulted from neuroinflammation induced by the neuroimmune system [2]. In research studies involving PD and AD, neuroinflammation has been reported to play the central role in the pathogenesis of these diseases. Neuroinflammation is regarded as an important feature of many neurodegenerative diseases such as multiple sclerosis (MS), narcolepsy, and autism [11].

Neuroinflammation stems from the immune system of the central nervous system (CNS) and comprises of a complex series of local immune processes constituting CNS cells such as neuron and glia, cytokines, pattern recognition receptors (PRRs), and peripheral immune cells in response to threats such as pathogens, tissue damage, abnormal stimulation, neurotoxins, infection, or injury. Neuroinflammation can assume a neuroprotective role or it can be counterproductive, causing damage to the nervous tissues. A persistent acute neuroinflammation can turn to a chronic neuroinflammation as it accumulates damage, bringing about neuronal degeneration. The effects or outcome of neuroinflammation has been indicated to be dependent on the time span of the inflammatory response and the activation state of microglia [12].

### 2.1. Role of Microglia in Neuroinflammation

Microglia are the innate immune cells in the CNS whereby it monitors and regulates the brain homeostasis, maintaining it under normal physiological conditions by purging pathogens as well as clearing dead cells through phagocytosis. Most notably, the microglia are critically involved in the neuroinflammatory response, serving as the initial indication of neuroinflammation when activated. The presence of pathogens, tissue damage, abnormal stimulation, neurotoxins, infection, injury, or any threats to the microenvironment activates microglia and thereafter the complex neuroinflammatory pathway [11] Macrophages can be activated into several distinct activation states, and the microglia functions differently according to the different activation states. The classical M1 type activation is the response to microorganism threats and is associated with cytotoxicity and inflammatory responses including the upregulation of proinflammatory cytokine expression. On the other hand, the M2 type activation is associated with immunoregulatory functions and tissue repair as well as wound healing and regeneration [13]. In response to an extensive and diverse array of microbial stimuli, the differential activation of microglia regulates neuroinflammation by inducing the release of proinflammatory mediators that favour the permeabilization of the blood brain barrier (BBB), which results in either neurotoxicity or neuroprotection [14]. Such stimuli are recognized by an array of receptors on microglia. The microglia activation states are named based on their effects on synaptic plasticity, neurogenesis, and learning and memory. Recently, data showed that microglial phenotypes switch from M2 to M1 depends upon the disease progression. M2 microglia is subdivided into three such as M2a, M2b, and M2c. M2a is involved to repair tissue-undergone damage by triggering anti-inflammatory and nerve growth factors. M2b regulates the deactivating phenotype and then produces anti-inflammatory mediators. M2c actively participates in phagocytosis and helps in cleaning process in the brain [15].

### 2.2. Role of Astrocytes in Neuroinflammation

Another important cell in the brain is astrocyte, which is considered to be a key regulator in the immunological system of both innate and adaptive immune responses at the time of stress or injury. The crucial role of astrocytes in inflammation is currently highlighted from both *in vivo* and *in vitro* findings [16]. Present literature has reported that intracellular signaling pathways are completely controlled by astrocytes during inflammation. Astrocyte responses might be beneficial for tissue repair process followed by injury. Besides, astrocytes play a role in the maintenance such as neurotransmitter uptake and gliotransmitter release [17]. Moreover, astrocytes are involved in cellular and molecular functions for degeneration, vascular signaling, and glial-neuronal interactions [18]. GFAP is the relevant marker for neuroinflammation when astrocytes are involved in AD [16]. Astrocytes also have interaction with cytokines, resulting in increased level of inflammatory markers. Proinflammatory signaling and reduced immune response due to high level of IL-10 induce deactivation of astrocytes [5].

## 3. Toll-Like Receptor on Motor Behaviour

PRRs are employed as sensors in the signal transduction of the innate immune system for the initial detection of microbial threats. Activated PRRs effectuate downstream signaling pathways which induce the innate immune responses by producing proinflammatory mediators, resulting in inflammation. One out of the several distinct classes of PRRs includes the TLRs family. TLRs are always found to be associated or involved in the induction of neuroinflammation in neurodegenerative diseases. TLRs are known to regulate the production of proinflammatory cytokines, which may contribute to further neuronal damage [19]. There are a total of 10 members of the TLRs family in humans; TLR1–TLR10 and 12 members in mice; TLR1–TLR9 and TLR11–TLR13. TLRs are expressed either on the exterior of microglia cells or to intracellular compartments such as the ER, endosome, lysosome, or endolysosome. Cell surface TLRs include TLR1, TLR2, TLR4, TLR5, TLR6, and TLR10, whereas intracellular TLRs include TLR3, TLR7, TLR8, TLR9, TLR11, TLR12, and TLR13 [20].

Toll-like receptor 4 (TLR4) particularly has been demonstrated in various studies to have a significant causal relationship with motor dysfunction in neurodegenerative conditions. TLR4 and other cell surface TLRs mainly detect and identify microbial membrane components, for example, lipids, lipoproteins, and proteins [20]. TLR4 activates upon stimulation of the Gram-negative lipopolysaccharide (LPS) which are known to trigger inflammation. The TLR4-LPS interaction has been found to result in physiological and behavioural changes including retardation of motor activity, loss of interest or pleasure, impaired cognitive function, and social withdrawal as well as reduced food and water intake [21]. TLR4 blockage with Tat-TLR4 interfering peptides injection was reported to suppress the event of sickness behaviour and exhibited absence of motoric and motivational effects of LPS-induced sickness [22]. Additionally, morphological changes in microglia and cytokine production that are typically induced by LPS were also blocked. Inhibition of TLR4 signaling prevents changes in behaviour and motivation caused by inflammatory stimulation, further suggesting the role and contribution of TLR4 in motor deficit.

Furthermore, suppression of TLR4 was also observed to reduce motor deficit conditions in neurodegenerative disorders and traumatic brain injury animal model. Feng et al., in 2016 [5], administered resatorvid, the TLR4 inhibitor TAK-242, in a rat subjected to controlled cortical impact injury. The result showed a neuroprotective effect through the inhibition of the TLR4-mediated pathway whereby the expression of TLR4 and its downstream signaling molecules, including MyD88, TRIF, NF-*κ*B, TNF-*α*, and IL-1*β*, was found to be significantly downregulated. However, a study by Zhu and colleagues [23] revealed a morphological-based analysis that linked TLR4 deficiency with thinning of the molecular layer of the cerebellum. The loss of TLR4 reduced the number of Purkinje cells (PCs) which are the sole output neurons of the cerebellar cortex, thus impairing motor function as PCs are responsible in regulating the function of the cerebellum which plays an essential role in balance and motor coordination [23].

## 4. Toll-Like Receptor 4 (TLR4) Neuroinflammatory Signaling Pathway

**Figure 1:**
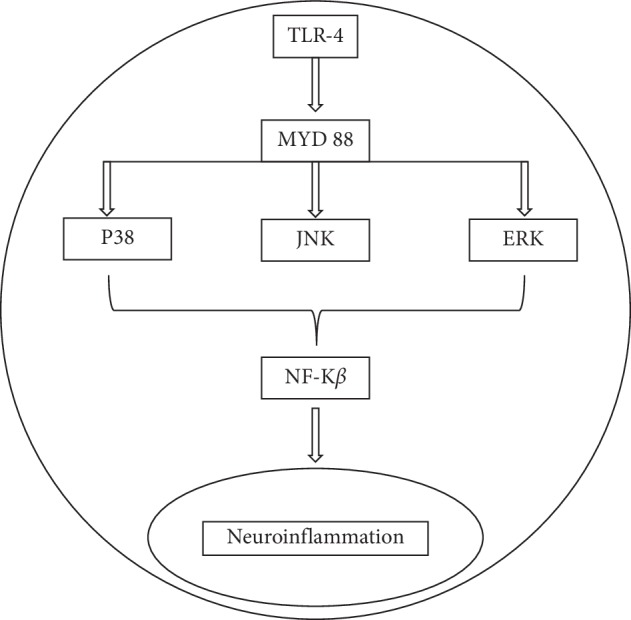
The schematic diagram shows the possible pathways to trigger neuroinflammation through toll-like receptor 4 in the brain.

Activation of TLRs initiates two signal transduction pathways, namely, the MyD88-dependent pathway and the MyD88-independent pathway. TLRs except TLR3 initiate intracellular signaling through ligand-induced dimerization of intracellular Toll-IL-1 receptor (TIR) domain [3]. TIR domains of TLR4 recruit TIR domain-containing adaptor proteins MyD88 and MAL of the MyD88-dependent pathway or TRIF and TRAM of the MyD88-independent pathway. The MyD88-dependent pathway activates IRAKs (IRAK1, IRAK2, and IRAK4) and TRAF6 that in turn activates TAK1. Subsequently, this leads to the activation of MAPKs (p38, JNK, and ERK1/2) and IKK pathways, resulting in NF-*κ*B activation which then induces the production of proinflammatory cytokines. The MyD88-independent pathway on the other hand activates TRIF and TRAM adaptor proteins which then recruit TBK1/IKK*ε* through the activation of TRAF3 (Figure 1). This then follows the activation of the transcription factor IRF3 in the nucleus leading to the production of type I interferons [3, 23]. Once LPS binds to TLR4 on the microglia surface, the signal transduction pathway is activated which in the end leads to NF-*κ*B activation. Activated NF-*κ*B functions to control DNA transcription, mediating the production of proinflammatory cytokines, chemokines, and inducible enzymes, namely, inducible nitric oxide synthase (iNOS) and COX-2 which are released from the microglia whereby all result in neuroinflammation [11, 24].

Previous studies had demonstrated that microglia in the brain region comprise an expression of TLR4 [25] and that the TLR4 activation activates microglia which in turn produces more proinflammatory factors such as tumor necrosis factor *α* (TNF*α*), interleukin-1*β* (IL-1*β*), and IL6 [26], resulting in a self-propelling and vicious cycle of neuroinflammation and neurodegeneration of dopamine neurons [27]. The production of proinflammatory cytokines is shown to be associated with reduced muscle mass and strength as well as affecting brain areas involved in motor coordination and fatigue [28]. To counter such reactions, IL-10, an anti-inflammatory cytokine, is produced by macrophages to suppress excess production of inflammatory cytokines and excessive inflammation [29]. Both NF-*κ*B and IL-10 play a functional role in the production and regulation of such proinflammatory cytokines, respectively. Proinflammatory cytokines produced as a result of TLR4 activation and NF-*κ*B triggering could affect the expression and regulation of neurotransmitters and receptors in the striatum and cerebellum in a way that possibly results in impaired motor functions.

## 5. Striatum and Cerebellum

The striatum is one of the main components of the basal ganglia which is involved in processes related to voluntary motor control. The striatum can be further divided into the dorsal striatum which consists of the caudate nucleus and putamen, and the ventral striatum which comprises of the nucleus accumbens and the olfactory tubercle. The striatum acts as the central glutamatergic and dopaminergic input receiving station and subsequently transmits these inputs to the rest of the basal ganglia. Within the striatum, the received inputs are projected onto two distinct classes of medium spiny neurons (MSNs) specified as the direct (striatonigral) and indirect pathway (striatopallidal) MSNs [30]. These two pathways differ whereby the direct pathway MSNs directly transmit inputs from the cortex and thalamus to the internal globus pallidus (GPi) and substantia nigra pars reticulata (SNr) while the indirect pathway MSNs receive input from the cortex and thalamus and indirectly transmit the outputs to SNr through the external GPe and subthalamic nucleus (STN). Moreover, the direct pathway MSNs express high levels of D1 dopamine while the indirect pathway MSNs have a high expression of D2 dopamine. Additionally, projections from the direct pathway MSNs are reported to mediate motor output, whereas projections from the indirect pathway MSNs impede motor output. The opposing activity of the two pathways is what regulates motor control [30].

Dysfunction of the connectivity or projections of the striatum is recognized as a notable cellular pathology in a number of motor and neurodegenerative diseases such as Parkinson's disease and Huntington's disease. Parkinson's disease (PD) is associated with a progressive decline in motor control. The causal circumstance of such decline is due to a dysfunction of the motor circuits within the striatum which is resulted from dopamine denervation in the dorsal striatum ascribable to the death of dopaminergic neurons in the SNr [30].

The cerebellum, also known as the “little brain,” is the major folded structure of the hindbrain. It consists of two cerebellar hemispheres whereby the cerebellar cortex comprise of three layers, which are the internal granular layer with granule cells, the middle Purkinje cell layer consisting of single row of Purkinje cells, and the molecular layer of cerebellum which is mainly made up of basket cells and stellate cells, two types of GABAergic interneurons. These cells receive excitatory synaptic inputs from granular neurons, and their axons make an inhibitory synapse with Purkinje cells. Axons of granule cells and the dendrites of Purkinje cells stretch out all the way into the molecular layer. Inputs from the cerebral cortex are transmitted to the cerebellum by mossy fibres which then excite the granule cells of the granular layer. The granule cells then specialize into parallel fibres which synapse into Purkinje cell dendrites, transmitting excitatory signals. At the same time, Purkinje cells also receive regulatory input through their axons from climbing fibres that stem from the inferior olive. Purkinje cells then sends an inhibitory signal to the deep cerebellar nucleus neurons that proceed toward the motor cortex. Concurrently, both mossy fibres and climbing fibres excite the deep cerebellar nucleus neurons. The output from deep cerebellar nucleus neurons thus depends on the overall inhibitory and excitatory stimulation [31].

**Figure 2:**
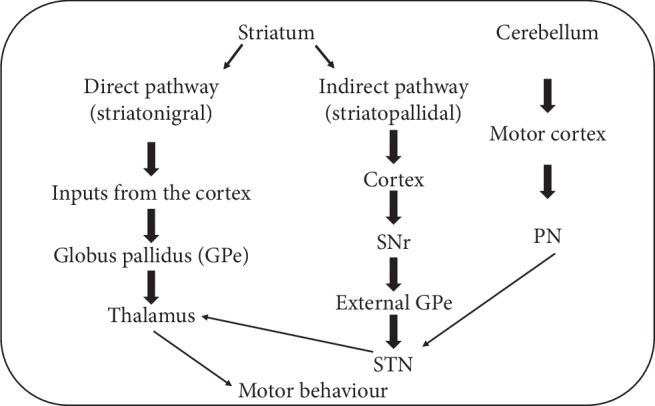
The schematic diagram shows the motor circuits from the striatum and cerebellum. The pathways connect the substantia nigra pars reticulata (SNr), subthalamic nucleus (STN), and cerebellum through the pontine nuclei (PN).

The cerebellum is critically involved in modulating various networks including voluntary motor control and cognition. Studies have showed a causal role of cerebellum dysfunction in motor impairment in a number of diseases such as PD and neurological movement disorders such as dystonia and multiple system atrophy (MSA). Mormina et al. [32] studied changes in the cerebellum in neurodegenerative diseases by using magnetic resonance imaging (MRI). All the aforementioned diseases are characterized with distinguished motor impairments and cerebellum dysfunction as their pathological hallmark. Loss in cerebellar volume was reported in PD patients with tremor due to cerebellar atrophy. Additionally, cerebellar hyperactivity was shown to be higher in PD patients. Similarly, atrophy of the middle cerebellar peduncles and volume loss of the middle and inferior cerebellar peduncles were also observed in MSA patients. Cerebellar atrophy and increased cerebellum activation together with the presence of cerebellar lesions and morphological cerebellar anomaly were observed in dystonia patients with hand stiffness. Dystonia is associated with continuous, unusual muscle contractions (Figure 2).

Another MRI study on the involvement of the cerebellum in the pathogenesis of ALS was conducted by [33]. ALS is a neurodegenerative disorder involving the motor neuron system in which it affects muscle contractions and progressively impact normal movement abilities. Motor impairments in ALS patients were linked with atrophy in the inferior cerebellum specifically the inferior lobules and vermis. Both the basal ganglia and the cerebellum interact with the cerebral cortex whereby the neuronal activity between the three structures is involved with parameters of movement [34]. In addition, past literatures reported that the primary brain regions most affected by inflammatory response include the basal ganglia, particularly the ventral striatum [35]. Both the striatum and cerebellum are selected as the areas of interest due to their involvement in motor control.

## 6. Neuroinflammation on Neurotransmitter's Receptors

Neurotransmitters are a diverse group of chemical compounds that are involved in the transmission of information in chemical synapses from the presynaptic site of one neuron to postsynaptic site of the adjacent neuron. Neurotransmitters from the presynaptic neuron diffuse into the synaptic cleft where they bind accordingly to their specific receptors to activate the respective signaling cascades. The neurotransmitters then either undergo the reuptake process by presynaptic transporter proteins and astrocytes or are degraded by specific enzymes that are present in the synaptic cleft. The resulting signaling cascade can elicit either an excitatory or inhibitory signal. Thus, neurotransmitters can be either excitatory or inhibitory in nature and are grouped accordingly based on structure and function [36]. Some of the neurotransmitter groups are as follows: acetylcholine, amino acids (glycine, glutamate and GABA (gamma aminobutyric acid)), amino acid derived amines (epinephrine, norepinephrine, dopamine, and serotonin), peptides (substance P and endorphins), purines (ATP), and gases (nitric oxide). Excitatory neurotransmitters include serotonin, acetylcholine, epinephrine, and norepinephrine, whereas inhibitory neurotransmitters include glycine and GABA. Studies had long demonstrated the involvement of various neurotransmitters in the proper functioning of motor neurons in the striatum and cerebellum [37, 38]. These studies involved the investigation of the functional relationship between neurotransmitters such as serotonin, GABA, dopamine, and glutamate with motor functioning.

## 7. Role of Gamma-Aminobutyric Acid (GABA) on Motor Behaviour and Neuroinflammation

GABA is a major inhibitory neurotransmitter in the CNS. The inhibitory process is regulated by inonotropic and metabotropic receptors which are located in presynaptic and postsynaptic regions [17]. Besides, GABA is one of the predominant inotropic receptors in the basal ganglia. The chloride conductance increased due to actions of its inhibitory role. Alteration in the GABAa receptor could cause motor deficits. Over activity of the striatal pathway suppresses dopamine in pallidus neuorons which is responsible for motor behavior in parkinsonian symptoms [39]. Drugs, for example, flumazenil, could facilitate motor behaviour interact with the GABAergic system which indicates that GABA has specific role in the modulation of motor behaviour [18]. The increased level of GABA tone in the cerebellum causes motor impairment [40]. Another study suggested that the GABA level increased in extracellular may reduce motor coordination [41]. Recent literatures reported that peripheral and central nervous system inflammation in diabetes or surgeries alters the GABAergic system, resulting in altered motor behaviour [42, 43]. This study suggested that motor coordination regulated by reduced status of neuroinflammation is related with normalization of the GABA neurotransmitter in the cerebellum [43]. The molecular mechanism results suggested by neuroinflammation could alter the GABAergic system in the cerebellum [43]. These studies clearly connected with neuroinflammation with GABAergic neurotransmission.

## 8. Role of Dopamine on Motor Behaviour and Neuroinflammation

Dopamine, unlike other neurotransmitters, can act as both inhibitory and excitatory neurotransmitter depending upon its location in the brain and which receptor it binds to. In dopamine receptors (DRs), Dopamine Receptor D1 (DRD1) mediates excitatory signal while Dopamine Receptor D2 (DRD2) mediates inhibitory signals. DRD1 is highly distributed in the striatum, nucleus accumbens, olfactory tubercle, cerebral cortex, and amygdala. Additionally, DRD2 is also highly communicated in the striatum, olfactory tubercle, and nucleus accumbens as well as in the substantia nigra pars compacta (SNc) and ventral tegmental area. The striatum acts as one of the main target region for dopamine involving the regulation of motor functions. Dopamine is critically involved in numerous brain circuits in the nervous systems associated with mediating motor control, feeding behaviour, cognitive functions, emotion, motivation, and reward [44].

Dopamine is generally known to be involved in the modulation of motor functions, and this has been stated and reiterated in numerous studies and articles. Neurotransmission and projection of dopamine from the substantia nigra to the striatum and to the cerebellum from the ventral tegmental area have been noted to influence the fine tuning of movements [45]. Nuclei in both SNc and the ventral tegmental area are reported to make up the major dopaminergic tracts [44]. The corticostriatal circuit expresses high levels of both Drd1 and Drd2, demonstrating the involvement of such receptors in controlling movement, thus justifying the selection of Drd1 and Drd2 in this study. Additionally, varied connection strength between striatum-cortical, striatum-cerebellar, and cortico-cerebellar motor influenced by imbalanced neurotransmission of dopamine were observed in Parkinson's patients with akinesia [46]. Subsequently, the production of cytokine during neuroinflammation is found to be involved in the alterations in dopamine neurotransmission whereby cytokines ultimately lead to decreased dopamine synthesis, thus decreasing dopamine function which could lead to neurodegeneration.

## 9. Role of Serotonin on Motor Behaviour and Neuroinflammation

Serotonin (5-hydroxytryptamine, 5-HT) acts upon excitatory transmission and operates as a mediator in inflammatory processes. 5-HT neurons are widely dispersed in the raphe nuclei of the brain stem such as the pons and medulla oblongata and additionally other brain regions, for example, the striatum, hippocampus, amygdala, cerebral cortex, thalamus, hypothalamus, and spinal cord [47]. Besides governing the regulation of critical physiological processes such as motor activity, sleep, body temperature, and pain, 5-HT is also significant in mediating endocrine and autonomic systems as well as emotional behaviour and cognitive function [48]. 5-HT has been reported to enhance and/or depress glutamate-mediated transmission as well as GABA-mediated transmission in structures controlling the movement [8]. 5-HT receptors, sorted into 7 families consisting of 5-HT1, 5-HT2, 5-HT3, 5-HT4, 5-HT6, and 5-HT7, mediate the serotonergic signal transduction. These 7 families are further broken down into 14 subtypes which are 5-HT1A, 1B, 1D, 1E, 1F, 5-HT2A, 2B, 2C, 5-HT3, 5-HT4, 5-HT5A, 5B, 5-HT6, and 5-HT7. Found at both pre- and postsynaptic membrane, the 5-HT receptors with the exception of 5-HT3 receptors which are ligand-gated ion channels are G protein-coupled receptors [47, 48].

Serotonin is generally involved in the mediation of motor behaviour through the cerebellum. The serotonin innervations from other motor structures also influence the cerebellum to modulate motor behaviour [15]. Hoxha et al. reported that parallel fibre-Purkinje cell (PF-PC) synapse is proposed as mechanisms for motor learning. The PF-PC synapse is finely modulated by several neurotransmitters including serotonin. In Rett syndrome, serotonin neurotransmission mainly participates in motor control through the help of the hippocampus and cerebellum [49]. Schizophrenia of the human cerebellum also shows serotonin 5‐HT (2A) receptor expression in Purkinje cells along with motor behaviour [50]. In the cerebellar atrophy 5-HT increases in the cerebellum related with alteration in motor coordination [51]. Previous studies reported that 5-HT1A role in cerebellar ataxia as well [52].

Various 5-HT receptors mediating 5-HT neurotransmission were reported in the regulation of extrapyramidal motor functions which are implicated in the pathophysiology of various neurological disorders. This is supported by the findings of the ameliorating effect of 5-HT1A receptors activation on antipsychotic-induced extrapyramidal side effects (EPS) and motor disabilities in animal models of Parkinson's disease in a study by 39. Specifically, 5-HT1A and 5-HT2A/2C receptors are amongst the multiple receptors that are of great significance in the modulation of motor disabilities in animal models of Parkinson's disease [47]. Past study of such findings include an experiment that demonstrated the weakening of L-DOPA-induced dyskinesia in 6-hydroxydopamine-lesioned rats through administration of mixed 5-HT1A/1B receptor agonist, eltoprazine [53]. Another study showed a decrement in tacrine-induced tremulous jaw movements in rats which are considered a primary motor symptom of tremor through the administration of 5-HT2A receptor inverse agonist and antagonist, ACP-103 [54]. Additionally, both 5-HT1A receptor agonist and 5-HT2A receptor inverse agonist and antagonist were shown to reduce L-DOPA-induced dyskinesia in MPTP-treated macaques. Serotonin has role in innate and adaptive immunity. Serotonin could trigger lymphocytes and monocytes which have an impact on the secretion of cytokines [55]. Recent literature reported that proinflammatory functions mainly active 5-HT2A receptors subtypes which inhibit TNF-*α* mediated inflammation (Yu, B). The animal studies showed that 5-HT2A blocks inflammatory response and prevents TNF-*α* activity (Nau).

Brain microglia expressed the mRNA of serotonin receptors. 5-HT2B receptor is expressed in microglia and supported for brain maturation [56]. From the literature, it is understood that serotonin has a main role in the modulation of neuroinflammation and motor behaviour.

## 10. Role of Glutamate on Motor Behaviour and Neuroinflammation

Glutamate is the most prevalent excitatory neurotransmitter in the CNS, having an extensive functional contribution in both the CNS and peripheral nervous system (PNS) processes as it is involved in various metabolic pathways. Present on glutamatergic neurons, glutamate execute glutamatergic signal transduction by binding to and, hence, activating both ionotropic and metabotropic glutamate receptors located on postsynaptic neurons. Regulation of glutamate is critical as unsuppressed glutamate release will result in glutamate dysregulation which poses excitotoxicity within the CNS. Such occurrence leads to neuronal damage and even neuronal death. Glutamate dysregulation has been well characterized in certain psychiatric, neurodevelopmental, and neurodegenerative disorders. The excitatory amino acid transporters (EAATs) hold the responsibility in preventing glutamate dysregulation by governing the release and reuptake of glutamate. Furthermore, glutamate transporters also contribute to learning, memory, and motor behaviour regulation. There are a total of five EAAT subtypes which are EAAT1 or GLAST (glutamate/aspartate transporter), EAAT2 (glutamate transporter-1), EAAT3 or EAAC1 (excitatory amino acid carrier-1), EAAT4, and EAAT5 [57].

Recently, it has been well studied that astrocytes play key role in the regulation of synaptic communication through modulating neurotransmitters and neuromodulators. It is reported that astrocytes control specifically and rapidly glutamate transmission [58]. Glutamate is a major excitatory neurochemical in the brain which is critical for maintaining normal CNS function. Glutamate and glutamine cycle are well mediated by astrocytes where released glutamate is recycled to glutamine in glial cells [59]. EAATs located in the neuron and astrocytes maintain glutamate release [60]. The excess of GLU is mainly regulated by GLU transporters on astrocytes [59]. Astrocytes regulate synaptic glutamate level by GLAST. The past and present animal studies reported knockdown or inhibition of GLAST results in increased level of GLU [61]. This caused motor impairment in rotarod. Astrocytes mainly involved in the mediation of pathological condition. The GLU and GABA are mostly regulated in the cerebellum by astrocytes. Neuroinflammation and glutamate toxicity play an important role in neurodegenerative process, resulting in elevated levels of inflammatory markers in ischemia and Parkinson's disease [62]. Besides, abnormal inflammatory mediates oxidative stress then may lead to glutamate excitotoxicity which plays an important role in pathogenesis [63]. Glial cells especially astrocytes are potentially involved in both glutamatergic and inflammatory process. Cytokine is well linked with neuroinflammatory process and glutamate-mediated toxicity. Glutamate released by neuronal vicious circle, microglia vicious circle, and astroglial vicious circle are regulated by glutamate transporters (Serafini).

Neurological disorders such as stroke, epilepsy, amyotrophic lateral sclerosis (ALS), Alzheimer's disease (AD), and Parkinson's disease (PD) exhibit alterations in the function or expression of glutamate transporters (EAATs) in their pathogenesis [64]. Zhang et al. [57] in their study showed that PD animal models exhibit a decreased expression and function of EAATs. EAATs especially EAAT1 are important in the maintenance of extracellular glutamate concentrations below glutamate excitotoxic levels, because high glutamate concentration results in glutamate neurotoxicity and subsequent dopamine neuronal death, movement disorder, and cognitive impairment [65]. Concentration of extracellular glutamate increases in the early stages of neuroinflammation due to microglia activation. A study by [66] demonstrated that fluctuation in neuronal glutamate transporter EAAT4 expression levels can alter the extrasynaptic glutamate signaling. Furthermore, both direct and indirect pathways of the corticostriatal circuit receive glutamatergic inputs that alter glutamate transmission in the dorsal striatum through NMDARs blockade which may contribute hyperactivity of motor function.

## 11. Future Perceptive

While the above studies provide valuable information regarding the potential associative mechanism between TLR, neuroinflammation and connection with neurotransmitters in the striatum- and cerebellum-mediated motor behaviour, there are still gaps in understanding the involvement and potential changes in functional neurotransmitter receptors and transporters that need to be investigated in order to know the complete mechanism of TLR4 activation in affecting motor behaviour. The known underlying pathway can provide alternative therapeutic treatment for existing neurological and motor neuron diseases. Therefore, this review was placed on the implication of the TLR toward the motor behaviour and associated neurotransmitter receptors and transporters using the animal model.
